# Declining Malaria Burden and Changing Species Distribution: A Twenty-Four-Year Retrospective Hospital-Based Study (2002–2025) in Mangaluru, India

**DOI:** 10.26502/fjhs.447

**Published:** 2026-10-05

**Authors:** Benudhar Mukhi, Chandan Kumar, Prince Kumar, D Pradeep, Mahesh Chandra Sahu, Anup Kumar R. Anvikar, Susanta Kumar Ghosh, Dipak Kumar Raj

**Affiliations:** 1 University of South Florida Health, Tampa, Florida, USA.; 2 ICMR-National Institute of Malaria Research, New Delhi, India.; 3 Department of Applied Zoology, Mangaluru University, Mangaluru, India.; 4 ICMR-Regional Medical Research Centre, Bhubaneswar, Odisha, India

**Keywords:** Antimalarial drug resistance, Epidemiology, follow-up surveillance, Malaria elimination, Mixed infection, *Plasmodium falciparum*, *Plasmodium vivax*, Treatment adherence, Southeast Asia

## Abstract

**Background:**

Malaria elimination is hindered by incomplete treatment and inadequate post- treatment follow-up, particularly among migrant and socioeconomically vulnerable populations. The increasing predominance of *Plasmodium vivax* further complicates elimination efforts.

**Methods:**

We conducted a 24-year retrospective hospital-based study (2002–2025) at Government Wenlock Hospital, Mangaluru, Karnataka, India, a tertiary health care center serving a historically malaria-endemic region characterized by rapid urbanization and a large migrant workforce. Suspected cases were clinically evaluated and confirmed by blinded microscopic examination of peripheral blood smears. Species distribution, parasitemia, treatment status, day-7 follow-up, and temporal and seasonal trends were analyzed using R and Python.

**Results:**

Among 345,544 suspected cases, 78,053 (22.59%) were microscopy-confirmed. Malaria positivity declined from 24.83% in 2002 to 0.35% in 2025. In 2025, *P. vivax* accounted for 66.7% of infections and *P. falciparum* for 33.3%; no mixed infections were detected. Parasitemia was highest in *P. falciparum*, followed by mixed and *P. vivax* infections. The proportion of *P. vivax* increased from 53.39% in 2005 to 86.95% in 2022, whereas *P. falciparum* declined from 45.15% to 10.81%. Although malaria cases were often concentrated during the post-monsoon period, the timing and magnitude of seasonal peaks varied substantially across years.

**Conclusions:**

Malaria declined markedly over the study period, but poor post-treatment follow-up and the increasing predominance of *P. vivax* remain important barriers to elimination. Strengthened patient tracking, treatment-completion monitoring, and species- specific surveillance are needed, particularly for migrant populations.

## Introduction

Malaria is a serious and widespread vector-borne infectious disease that affects millions of people around the world. Despite decades of dedicated efforts to control and eradicate it, malaria continues to cause significant illness and death among vulnerable populations [1]. According to the World Health Organization (WHO), an estimated 282 million malaria cases and approximately 610,000 malaria-related deaths occurred globally in 2024 [1], with the greatest burden concentrated in low- and middle-income countries [2]. Although significant progress has been achieved over the past two decades, malaria continues to pose a major public health challenge, particularly in tropical and subtropical regions where environmental conditions favor sustained transmission [3, 1]. The WHO Southeast Asia Region (SEARO) has experienced a substantial decline in malaria burden, with reported cases decreasing by more than 80% since 2000 [4]. India has contributed significantly to these achievements and remains committed to achieving malaria elimination by 2030 under the National Framework for Malaria Elimination [5, 6]. Nevertheless, persistent transmission in several endemic areas continues to hinder elimination efforts [7]. Urban malaria remains a cause for concern because rapid urbanization, population movement, construction activities, inadequate water management, and the expansion of *Anopheles stephensi* breeding habitats create favorable conditions for ongoing transmission [8, 9].

Over the past two decades, malaria epidemiology in India has undergone important changes. While the burden of *Plasmodium falciparum* has declined in many regions, *Plasmodium vivax* has emerged as increasingly dominant [10]. Unlike *P. falciparum*, *P. vivax* presents unique challenges to elimination due to its ability to form dormant liver-stage hypnozoites, which can result in relapses that sustain transmission despite successful treatment of blood-stage infection [11]. Furthermore, asymptomatic and low-density infections frequently escape routine diagnostic detection, contributing to a hidden parasite reservoir that may maintain a local residual transmission [12].

Effective malaria elimination requires not only prompt diagnosis and treatment but also adequate treatment completion and post-treatment monitoring [13]. Incomplete antimalarial therapy, treatment interruption, poor adherence, and loss to follow-up may compromise treatment effectiveness and reduce the accuracy of surveillance systems. These challenges are particularly pronounced among migrant laborers and socioeconomically vulnerable populations, who often face barriers to healthcare access, continuity of treatment, and follow- up assessment [14, 15]. In addition, mixed-species infections remain difficult to diagnose using routine microscopy and may complicate clinical management when species identification is incomplete [16]. Long-term epidemiological surveillance provides valuable insights into changing transmission dynamics, species distribution, treatment practices, and operational barriers to malaria control [17]. Such data are essential for identifying gaps in current elimination strategies and informing evidence-based public health interventions [18]. Mangaluru, a rapidly developing coastal city in southwestern India, represents an important urban malaria setting characterized by substantial population mobility, favorable climatic conditions for vector breeding, and a history of persistent malaria transmission [9]. The present study analyzes 24 years of clinical and epidemiological malaria surveillance data collected between 2002 and 2025 at a major tertiary care referral hospital in a Southeast Asian region. Specifically, we examined temporal trends in malaria incidence, species distribution, parasitemia patterns, treatment delivery, and post-treatment follow-up. By characterizing long- term changes in malaria epidemiology and identifying operational gaps in case management, this study provides insights relevant to malaria control and elimination efforts in India and other similar endemic settings.

## Methods

### Selection of Study Site, Design, and Population

This retrospective hospital-based study was conducted in Mangaluru, a coastal city in Karnataka State, southwestern India, between January 2002 and December 2025. Mangaluru is situated between the Arabian Sea and the Western Ghats and experiences a tropical monsoon climate characterized by high humidity, annual rainfall exceeding 3,700 mm, and a mean annual temperature of ~ 27°C [9, 19]. These environmental conditions, together with rapid urbanization, construction activities, water storage practices, and the presence of suitable mosquito breeding habitats, contribute to sustained malaria transmission in the region [9, 20]. The study was conducted at Wenlock District Government Hospital, the largest public tertiary- care hospital in Mangaluru and a major referral center for malaria diagnosis and treatment in the surrounding districts [21, 22, 23]. The hospital provides free healthcare services to economically disadvantaged populations and serves a diverse patient population from urban, peri-urban, and rural communities throughout coastal Karnataka and neighboring states, which have significantly higher malaria cases than other regions of the state [9, 21]. Patients presenting with clinical features suggestive of malaria and an axillary temperature >37.5°C were screened for malaria infection. Malaria diagnosis was confirmed by expert microscopic examination of Giemsa-stained peripheral blood smears [21]. Rapid diagnostic tests (RDTs) were introduced as supplementary diagnostic tools beginning in 2013 in accordance with national malaria control guidelines [24, 25]. Following the introduction of rapid diagnostic tests (RDTs) in 2013 to facilitate timely case detection, microscopy remained the primary method for confirming malaria infection and determining parasite species. Therefore, the introduction of RDTs may have improved case ascertainment, particularly by increasing diagnostic access or sensitivity, but did not alter the microscopy-based criteria used to define confirmed cases in this study. A total of 78,053 patients across different age groups, predominantly children and migrant workers, with microscopically confirmed malaria were included in the study. The study population comprised 11,704 individuals aged ≤18 years (15.0%), 46,837 adult males (60.0%), 19,222 adult females (24.6%), and 290 transgender individuals (0.4%). The study population represented diverse ethnic, linguistic, and socioeconomic backgrounds. Participants originated primarily from Karnataka and neighboring Indian states and included migrant laborers employed in construction, hospitality, domestic service, security, and other labor-intensive occupations [9]. This heterogeneous population reflects the demographic composition of Mangaluru, a rapidly expanding urban centre that attracts substantial internal and cross-border migration., both of which may influence malaria transmission dynamics and healthcare utilization patterns.

### Ethical Approval and Consent to Participate

Blood sample processing, parasitemia assessment, malaria diagnosis, and clinical data collection were conducted as part of routine patient care in accordance with institutional protocols and applicable Government of India guidelines throughout the study period (2002–2025). The retrospective review and analysis of de-identified patient data were approved by the Institutional Ethics Committee of the ICMR–National Institute of Malaria Research, New Delhi, India (Ref. No. ECR/NIMR/EC/12/39), and the Institutional Ethics Committee of Kasturba Medical College, Manipal Academy of Higher Education, Mangaluru, Karnataka, India (Ref. No. IEC KMC MLR 16/49). Given the retrospective nature of the study and the use of de-identified data, the requirement for individual informed consent was waived by the respective ethics committees. The study was conducted in accordance with the principles of the Declaration of Helsinki and applicable national ethical guidelines. Patient confidentiality was maintained throughout the study, and all data were de-identified before analysis.

### Sample Collection and Evaluation of Parasitemia

Between 2002 and 2025, a total of 345,544 patients presenting with suspected malaria were screened at our study site. Parasitemia was assessed on the day of hospital presentation using peripheral blood smears. Three experienced blinded microscopists independently examined all slides. Discrepant microscopy results among the three microscopists were resolved by repeat examination of the blood smear and consensus review; when disagreement persisted, the final diagnosis was based on review by the most experienced microscopist. Parasite densities were quantified according to standardized procedures, and the results are summarized in Table 1. Of these, 78,053 (22.59 %) were microscopically confirmed malaria cases. Among the confirmed cases, 56,621 (72.54%) were infected with *Plasmodium vivax*, 19,346 (24.78%) with *Plasmodium falciparum*, and 2,086 (2.67%) had mixed (*P. vivax* + *P. falciparum*) infections. Patients with suspected malaria underwent clinical evaluation followed by microscopic examination of peripheral blood smears for laboratory confirmation (Table 2 and Table 3). Thick and thin blood smears were prepared from finger-prick blood samples and stained with 4% Giemsa solution. After air-drying, slides were examined using a Zeiss Primo Star light microscope (Zeiss, Germany) at 1000× magnification (100× oil immersion objective and 10× eyepiece) [21]. Parasite density (parasitemia) was determined according to standard microscopic methods [22]. Clinical assessments were repeated after treatment, and follow-up blood smears were examined on day 7 to assess treatment outcomes and guide further clinical management. Because the study was based on malaria episodes rather than unique individuals, a patient could potentially contribute more than one malaria episode during the study period. Patients included in follow-up were confirmed to be malaria-negative after treatment, and patient identification records were retained for up to six months to facilitate recognition of repeat presentations. Accordingly, a subsequent malaria-positive presentation following documented parasite clearance was considered a new malaria episode for the purposes of this retrospective analysis. However, because molecular genotyping was not performed, recrudescence, relapse, and reinfection could not be definitively distinguished.

### Case Management and Antimalarial Treatment Strategies Supporting Malaria Elimination

Prompt diagnosis and effective treatment were cornerstones of malaria control and elimination efforts throughout the surveillance period [26]. Species-specific antimalarial regimens were administered in accordance with national malaria treatment guidelines to ensure rapid parasite clearance, prevent disease progression, and interrupt local transmission. Patients with uncomplicated *Plasmodium vivax* malaria were treated with oral chloroquine for 3 days, followed by primaquine (0.25 mg/kg/day) for 14 days to achieve radical cure by eliminating dormant liver-stage hypnozoites responsible for relapse [24]. Uncomplicated *Plasmodium falciparum* infections were treated with artemisinin-based combination therapy (ACT; artesunate, sulfadoxine, and pyrimethamine) for 3 days, together with a single dose of primaquine administered on day 2 to reduce gametocyte carriage and block onward transmission [24]. Patients with mixed (*P. falciparum* + *P. vivax*) infections received ACT for 3 days, combined with a 14-day course of primaquine, to ensure clearance of both blood-stage parasites and latent hepatic forms [24]. Treatment adherence was actively monitored through clinical follow-up and patient counseling, while repeat blood smear examinations were performed to confirm parasite clearance and therapeutic response. Early diagnosis, prompt initiation of effective antimalarial therapy, and systematic follow-up were integral components of the regional malaria control program. Collectively, these interventions played a critical role in the progressive decline of malaria incidence and the findings consistent with substantial progress toward malaria elimination. Primaquine was not administered to patients with known glucose-6-phosphate dehydrogenase (G6PD) deficiency or a documented family history of G6PD deficiency.

### Challenges in Treatment Adherence and Case Follow-up

Despite the availability of effective antimalarial therapies, several operational and behavioral factors hindered optimal case management throughout the surveillance period. Prior to medication dispensation, laboratory personnel provided counseling on treatment adherence, follow-up blood smear examination, and the importance of clinical review. However, patient compliance varied considerably, with some individuals initiating treatment independently without medical supervision and others failing to attend scheduled follow-up visits. In severe cases requiring hospitalization, treatment interruption occurred when patients absconded before completing therapy, often due to financial concerns, fear of hospitalization, or lack of social support. Additional challenges, including unreliable contact information, patient non-cooperation, and the absence of caregivers, further complicated case tracking and continuity of care. These barriers increased the risk of incomplete treatment, relapse or recrudescence, and persistence of infectious reservoirs, highlighting the significant operational challenges encountered in achieving and sustaining malaria elimination.

### Statistical Analysis

Statistical analyses were performed using the complete annual malaria surveillance dataset collected between 2002 and 2025. Annual malaria positivity was calculated as the number of microscopy-confirmed malaria cases divided by the total number of blood smears examined. Species-specific proportions were calculated as the numbers of *Plasmodium vivax*, *P. falciparum*, and mixed (*P. vivax* + *P. falciparum*) infections divided by the total number of malaria-positive cases in each year. Temporal trends in malaria positivity were evaluated using grouped binomial logistic regression, with calendar year modeled as a continuous predictor and the annual numbers of malaria-positive and malaria-negative blood smears as the binomial outcome. Temporal changes in species composition were evaluated similarly by modeling the annual number of infections attributable to each malaria species relative to the total number of malaria-positive cases. To account for heterogeneity among annual surveillance observations, heteroskedasticity-robust standard errors were used for inference. Because linear regression-based trend tests are influenced by the substantially greater numbers of malaria cases observed during the earlier high-transmission years, Spearman’s rank correlation was additionally used as an unweighted nonparametric assessment of monotonic trends across the 24 annual observations. Fisher’s exact test was used for selected two-group comparisons when cell counts were small. All statistical tests were two-sided, and a *P* value <0.05 was considered statistically significant. Pearson correlation coefficients were used only for descriptive assessment of relationships among surveillance indicators. Correlations between total malaria-positive cases and individual species counts were interpreted cautiously because species-specific counts are mathematical components of the total positive-case count (Table 1 & Figure 2).

## Results

### Long-term Decline in Malaria Positivity

Between 2002 and 2025, a total of 345,544 blood smears were examined, of which 78,053 (22.59%) were positive for malaria (Figure 1A, B, C). Malaria positivity declined markedly over the surveillance period, from 24.83% (2,587/10,417) in 2002 to 0.35% (12/3,402) in 2025, representing an approximately 98.6% relative reduction. Grouped binomial regression demonstrated a highly significant temporal decline in malaria positivity. For each additional calendar year, the odds of a blood smear being malaria-positive decreased by ~ 9.2% (OR per year = 0.908; 95% CI, 0.878–0.939; P < 0.0001). The temporal decline was independently supported by Spearman’s rank correlation (ρ = −0.891; P < 0.0001), indicating a strong monotonic reduction in annual malaria positivity.

A direct comparison between the beginning and end of the surveillance periods showed that the odds of malaria positivity in 2025 were ~ 99% lower than in 2002 (OR = 0.011; 95% CI, 0.006–0.019; Fisher’s exact test, P < 0.0001). Malaria positivity reached very low levels during the last 4 years of surveillance, declining to 0.79% in 2022, 0.10% in 2023, 0.34% in 2024, and 0.35% in 2025, respectively. This trend is consistent with a transition from high endemicity toward interruption of sustained local transmission.

### Long-term Change in Malaria Species Composition

Across the 2002–2025 surveillance period, *P. vivax* accounted for 56,621 cases (72.54% of all positive cases), *P. falciparum* for 19,346 cases (24.78%), and mixed infections for 2,086 cases (2.67%) (Figure 2A).. Count-weighted binomial regression demonstrated a significant long-term increase in the proportional contribution of *P. vivax* (Figure 2D). The odds that a malaria-positive infection was attributable to *P. vivax* increased by ~ 8.3% per calendar year (OR per year = 1.083; 95% CI, 1.037–1.131; P = 0.0004). Conversely, the proportional contribution of *P. falciparum* declined significantly over time (Figure 2D). Each additional calendar year was associated with an approximately 10.2% reduction in the odds that a malaria-positive infection was *P. falciparum* (OR per year = 0.898; 95% CI, 0.859–0.940; P < 0.0001). These findings demonstrate a substantial epidemiological transition from a relatively greater contribution of *P. falciparum* during the earlier high- transmission period toward predominance of *P. vivax* among residual malaria infections.

However, when each calendar year was weighted equally using Spearman’s rank correlation, the monotonic trend in species percentages was weaker over the complete 2002–2025 interval. The correlation between calendar year and the annual proportion of *P. vivax* was ρ = 0.295 (P = 0.162). In contrast, the correlation between calendar year and the annual proportion of *P. falciparum* was ρ = −0.344 (P = 0.099). This difference between the regression and rank-based analyses reflects the extremely small number of malaria cases during 2022–2025, when year- specific species percentages became unstable (Figure 2C). For example, only 13, 43, and 12 malaria-positive cases were recorded in 2023, 2024, and 2025, respectively. Therefore, the count-weighted regression estimates provide a more informative measure of the long-term change in species composition, whereas percentages from individual low-incidence years should be interpreted cautiously (Figure 2D).

### Temporal Pattern of Mixed-species Infections

Mixed *P. vivax + P. falciparum* infections accounted for a relatively small proportion of the total malaria burden but showed substantial interannual variability (Figure 2C). Count- weighted binomial regression produced a statistically significant positive temporal coefficient (OR per year = 1.137; 95% CI, 1.066–1.212; P < 0.0001). However, the annual percentage of mixed infections did not demonstrate a significant monotonic trend when each year was given equal weight (Spearman ρ = 0.103; P = 0.633). Inspection of the annual data indicated that this discrepancy resulted from a temporary concentration of mixed infections midway through the surveillance period rather than a sustained, progressive increase. Mixed infections increased particularly during 2014–2016, reaching 11.55% of malaria-positive cases in 2015, before declining again during subsequent years. Accordingly, mixed infections are most appropriately described as exhibiting a transient mid-surveillance spike rather than a continuous long-term upward trend (Figure 2C).

The difference between the statistically significant count-weighted regression trends for species composition and the nonsignificant Spearman’s correlation reflects their different weighting of annual observations. Regression gives greater statistical weight to years with thousands of confirmed infections, whereas Spearman’s correlation assigns equal weight to high-incidence years and the final years with only 12–46 cases. Accordingly, the regression analysis more precisely characterizes the overall epidemiological transition, while the Spearman analysis highlights the instability of percentages in the final near-elimination years because of very small denominators.

### Treatment Coverage, Treatment Gaps, and Challenges to Malaria Elimination

Treatment coverage remained consistently high throughout the surveillance period, with the number of treated cases peaking in 2005 and subsequently declining in parallel with the decreasing malaria burden. Nevertheless, a small but persistent subset of laboratory-confirmed cases failed to initiate treatment across all infection categories, with *P. vivax* accounting for the largest proportion of untreated cases. Operational factors included delayed collection of laboratory results, patient attrition before medication dispensing, work-related constraints, limited health awareness, and difficulties in patient follow-up. These untreated infections represented potential reservoirs for continued transmission, underscoring the importance of strengthening case-management systems and ensuring universal access to prompt treatment.

### Follow-up Compliance and Post-treatment Monitoring

Follow-up assessment after antimalarial treatment varied across the study. Follow-up rates declined from 24.16% in 2002 to approximately 10% by 2006 and remained low for much of the surveillance period. A temporary improvement was observed between 2017 and 2019, culminating in the highest follow-up rate of 31.86% in 2019. However, follow-up compliance subsequently declined, reaching only 4.10% in 2021. Poor follow-up represented a major operational challenge because it limited the ability to confirm parasite clearance, detect treatment failure, monitor relapse, and assess transmission-blocking effectiveness. However, no recurrence of malaria or detectable malaria parasitemia was observed among individuals who complied with follow-up in the study population. Strengthening post-treatment surveillance remains essential for sustaining malaria elimination and preventing the re- establishment of local transmission.

## Discussion

### Transition from Hyperendemicity to Very Low Residual Malaria Burden

This 24-year passive hospital-based study documents Mangaluru’s transition from a hyperendemic malaria setting toward interruption of local transmission. The long-term decline likely reflects the cumulative contribution of enhanced diagnostic capacity, effective antimalarial treatment policies, strengthened vector control, and improved surveillance. Despite periodic fluctuations in transmission intensity, the sustained downward trajectory demonstrates the value of integrated malaria-control strategies for moving endemic regions toward elimination.

### Changing Epidemiology and the Emergence of a Predominantly Plasmodium vivax Landscape

One of the most important findings of this study was the progressive shift in species composition during the very low residual malaria burden phase. Although both *Plasmodium falciparum* and *Plasmodium vivax* declined in absolute numbers, *P. vivax* increasingly dominated the residual malaria burden. By 2022, nearly 87% of all confirmed malaria cases were attributable to *P. vivax*. Similar epidemiological transitions have been reported in several malaria-endemic regions approaching elimination, where control measures successfully reduce *P. falciparum* transmission while the biological characteristics of *P. vivax*, particularly hypnozoite-mediated relapse, sustain residual transmission [27, 28].

The increasing proportional contribution of *P. vivax* highlights the importance of radical cure strategies and effective follow-up [29]. Failure to complete primaquine therapy may allow latent liver-stage parasites to persist, contributing to recurrent infections and continued transmission [29, 30]. Consequently, elimination programs must prioritize interventions that specifically target *P. vivax* reservoirs to prevent the re-establishment of local transmission.

### Clinical Significance of Mixed Infections

Although mixed infections represented a relatively small proportion of total malaria cases, their clinical and operational significance was substantial. Patients with mixed infections exhibited a higher frequency of severe clinical manifestations, including hypertension, gastrointestinal symptoms, and respiratory complications, compared with mono-infections. These findings suggest that mixed infections may contribute disproportionately to malaria-associated morbidity [31]. Accurate identification of mixed infections remains a persistent challenge in routine microscopy-based surveillance systems [16]. Misclassification can result in inappropriate treatment [32], increased risk of relapse or recrudescence, and potential selection of drug- resistant parasites. The integration of molecular diagnostic approaches, including nested PCR and quantitative PCR, could improve the detection of mixed and submicroscopic infections, particularly during the very low residual malaria burden phase, when transmission intensity is low.

### Treatment Adherence and Operational Barriers to Elimination

A major challenge identified during the surveillance period was incomplete treatment adherence and poor follow-up compliance. Although treatment coverage remained high, a proportion of laboratory-confirmed cases failed to initiate therapy, did not complete prescribed regimens, or were lost to follow-up. In some instances, patients requiring hospitalization left healthcare facilities before completion of treatment. These operational gaps have important implications for malaria elimination. Incomplete antimalarial therapy exposes parasites to subtherapeutic drug concentrations, facilitating the selection and spread of resistant strains. Mutations in genes associated with antimalarial resistance, including *Pfcrt*, *Pfmdr1*, and *Pfk13*, have been reported in endemic regions [33, 34] and represent an ongoing threat to malaria control. Furthermore, inadequate follow-up limits the ability to identify early and late treatment failures, detect relapses, and confirm parasite clearance [35, 36, 37]. Socioeconomic factors played a significant role in treatment interruption. Migrant laborers and daily-wage earners frequently prioritized employment obligations over follow-up visits due to potential income loss [38]. Similar challenges have been documented throughout malaria- endemic regions and continue to impede progress toward elimination.

### Seasonal Transmission Dynamics and Public Health Implications

Heatmap analysis revealed considerable interannual variability in malaria transmission, although infections were consistently concentrated during post-monsoon months. Seasonal peaks generally occurred between September and December (Figure 3), corresponding to favorable environmental conditions for mosquito breeding and parasite development [39, 40, 41]. However, substantial year-to-year variation indicates that transmission is influenced by a complex interaction of climatic, ecological, and human behavioral factors (Figure 3). Understanding these temporal patterns is critical for optimizing malaria control strategies. Vector control interventions, including indoor residual spraying and insecticide-treated net distribution, should be strategically implemented before anticipated transmission peaks [42]. Climate-informed surveillance systems may further enhance preparedness by identifying periods of elevated transmission risk.

### Correlations Among Annual Surveillance Measures

Pearson correlations among annual numbers of blood smears examined and malaria cases by species. P. vivax case counts were strongly correlated with total malaria-positive case counts (r = 0.96), whereas the correlation between P. falciparum and mixed-infection case counts was weaker (r = 0.23, Figure 4). Because species-specific cases contribute to the total case count, correlations with that total partly reflect this mathematical relationship and should not be interpreted as evidence of a causal association.

### Impact of Human Mobility and the COVID-19 Pandemic

The temporary decline in malaria cases observed during the COVID-19 pandemic provides important insights into the relationship between human mobility and malaria transmission. Travel restrictions and the reverse migration of laborers likely reduced opportunities for exposure and interrupted local transmission chains. Similar observations have been reported from other endemic regions, highlighting the role of population movement in shaping malaria epidemiology [43]. The decline during the COVID-19 period coincided with major changes in population mobility; however, the present observational dataset cannot determine the extent to which mobility restrictions contributed to the reduction.

### Strengthening Surveillance for Sustained Elimination

As malaria incidence approaches zero, surveillance systems become increasingly important for identifying residual transmission foci and preventing reintroduction. The strong correlations observed between diagnostic testing volume, malaria positivity, and species-specific case detection underscore the value of maintaining robust surveillance infrastructure even after transmission declines. Future elimination efforts should incorporate molecular diagnostics, sentinel drug-resistance monitoring, therapeutic efficacy studies, and active case detection to identify low-density and asymptomatic infections. Enhanced surveillance will be essential to detect imported cases, monitor residual parasite reservoirs, and respond rapidly to potential outbreaks.

### Limitations

Several limitations should be considered when interpreting these findings. The study relied primarily on microscopy-based diagnosis, which may underestimate mixed infections and low- density parasitemia. Follow-up data were incomplete for a substantial proportion of patients, limiting assessment of treatment outcomes and relapse rates. In addition, the study did not systematically quantify socioeconomic and behavioral factors influencing treatment adherence. Nevertheless, the large sample size, extended surveillance period, and comprehensive clinical dataset provide valuable insights into malaria epidemiology during the transition from control to elimination.

## Conclusions

This 24-year hospital-based surveillance study documents a substantial decline in malaria burden in Mangaluru, India, with progression from hyperendemic transmission to very low residual malaria burden and interruption of sustained local transmission. As malaria declined, *P. vivax* became the predominant species among residual infections, emphasizing the importance of radical cure and relapse prevention. Persistent gaps in treatment initiation and post-treatment follow-up, particularly among mobile and socioeconomically vulnerable populations, remained important operational challenges. Sustaining elimination will require sensitive surveillance for imported, mixed, and low-density infections; effective case management and follow-up; monitoring of antimalarial efficacy and resistance; and continued vector-control measures to prevent re-establishment of local transmission. The Mangaluru experience provides useful evidence for other endemic regions transitioning from malaria control to elimination.

## Figures and Tables

**Figure 1:**
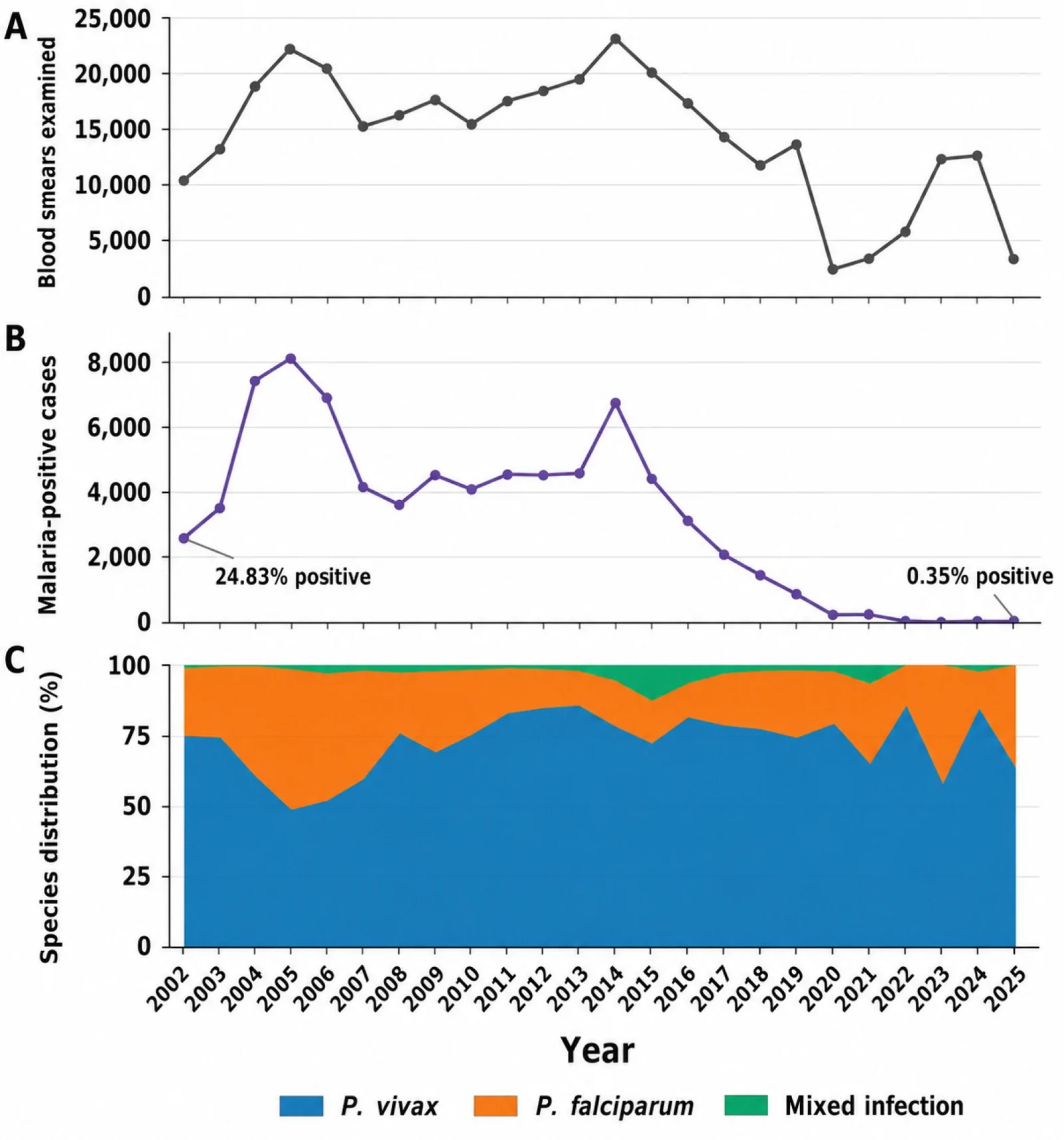
Annual Trends in Malaria Screening, Malaria-positive Cases, and Species Distribution, 2002–2025. (A) Annual number of blood smears examined for suspected malaria at Wenlock District Government Hospital, Mangaluru, India. (B) Annual number of microscopy-confirmed malaria-positive cases. Malaria positivity declined from 24.83% in 2002 to 0.35% in 2025. (C) Annual proportional distribution of *Plasmodium vivax*, *P. falciparum*, and mixed (*P. vivax* + *P. falciparum*) infections among confirmed cases. During the 24-year surveillance period, 345,544 blood smears were examined, of which 78,053 (22.59%) were positive for malaria. Species percentages in panel C were calculated using the total number of species- classified malaria-positive cases in each year as the denominator.

**Figure 2:**
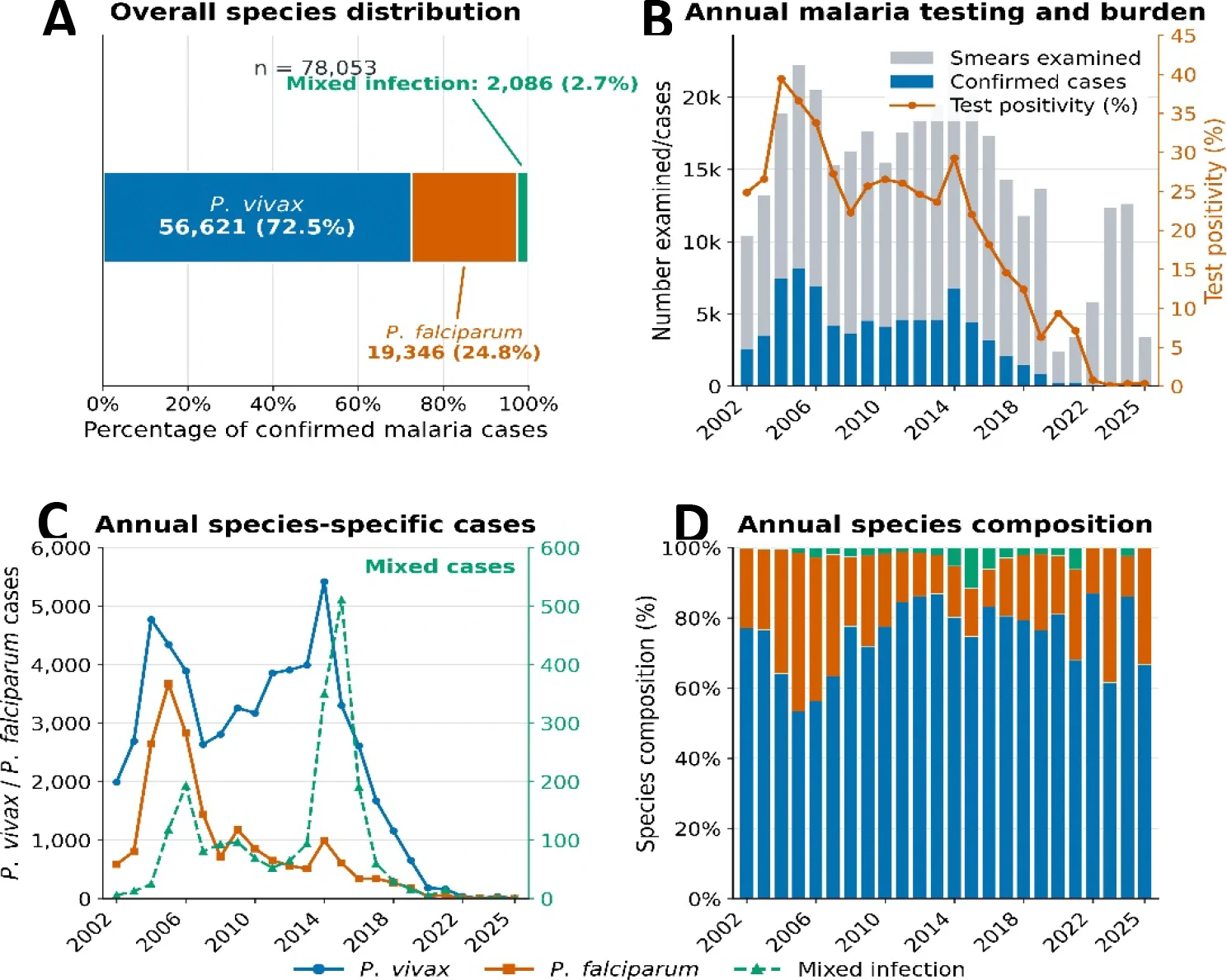
Clinical Malaria Burden and Species Distribution, 2002–2025. (A) Overall distribution of *Plasmodium vivax*, *P. falciparum*, and mixed infections among 78,053 confirmed malaria cases. (B) Annual numbers of blood smears examined and confirmed malaria cases, with the corresponding test-positivity rate. (C) Annual species-specific case counts; mixed infections are displayed on the secondary y-axis. (D) Annual proportional distribution of *P. vivax*, *P. falciparum*, and mixed infections among confirmed cases. Data represent 345,544 blood smears examined during the 24-year study period.

**Figure 3:**
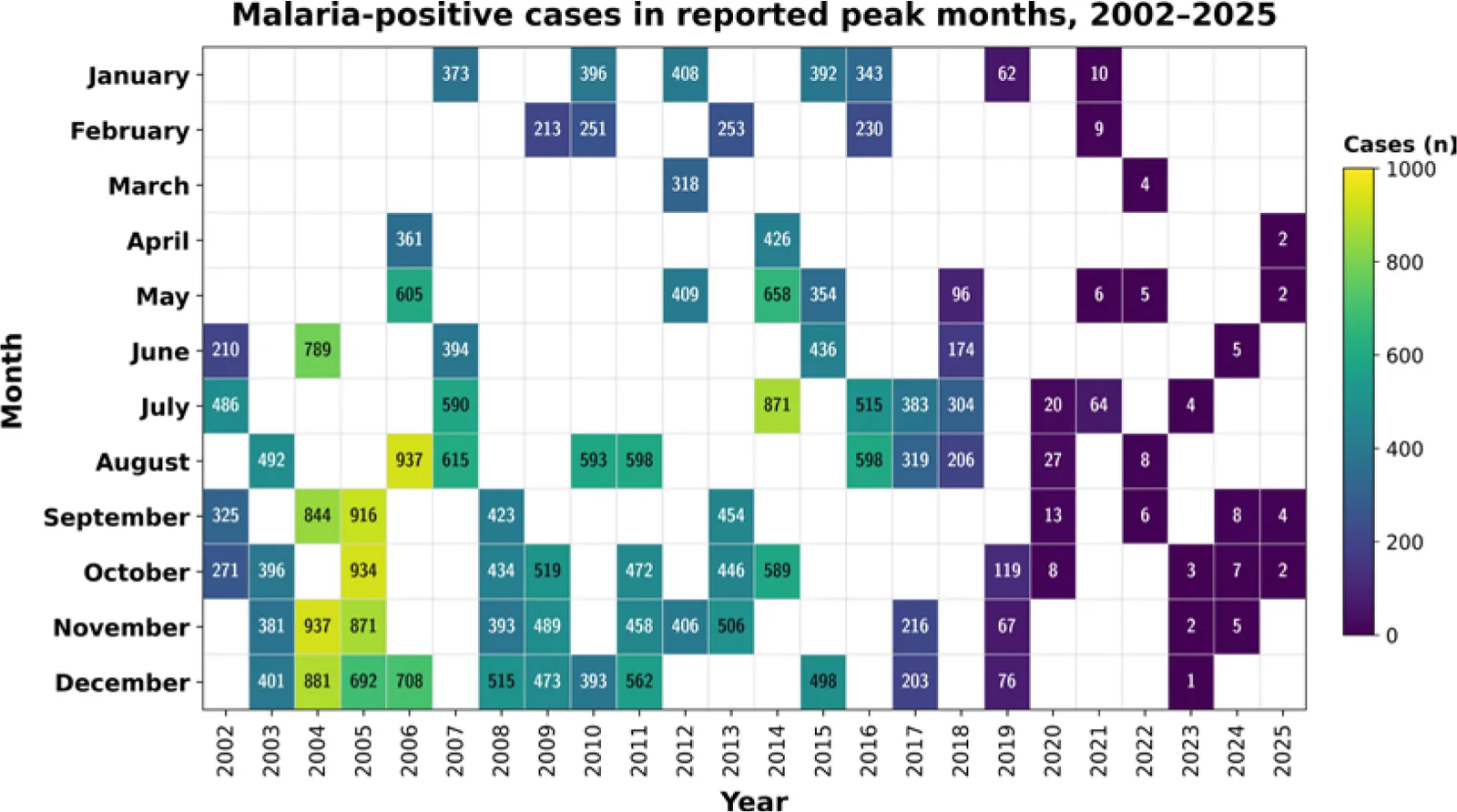
Annual Distribution of Malaria-positive Cases During Reported Peak Months, 2002–2025. The heatmap presents the number of malaria-positive cases recorded during the reported peak- transmission months for each year at Wenlock District Government Hospital, Mangaluru, India. Numbers within the coloured cells indicate malaria-positive cases (*n*), while colour intensity represents the corresponding case burden according to the scale. White cells indicate months that were not identified or reported as peak months for the corresponding year and should not be interpreted as months with zero malaria cases.

**Figure 4:**
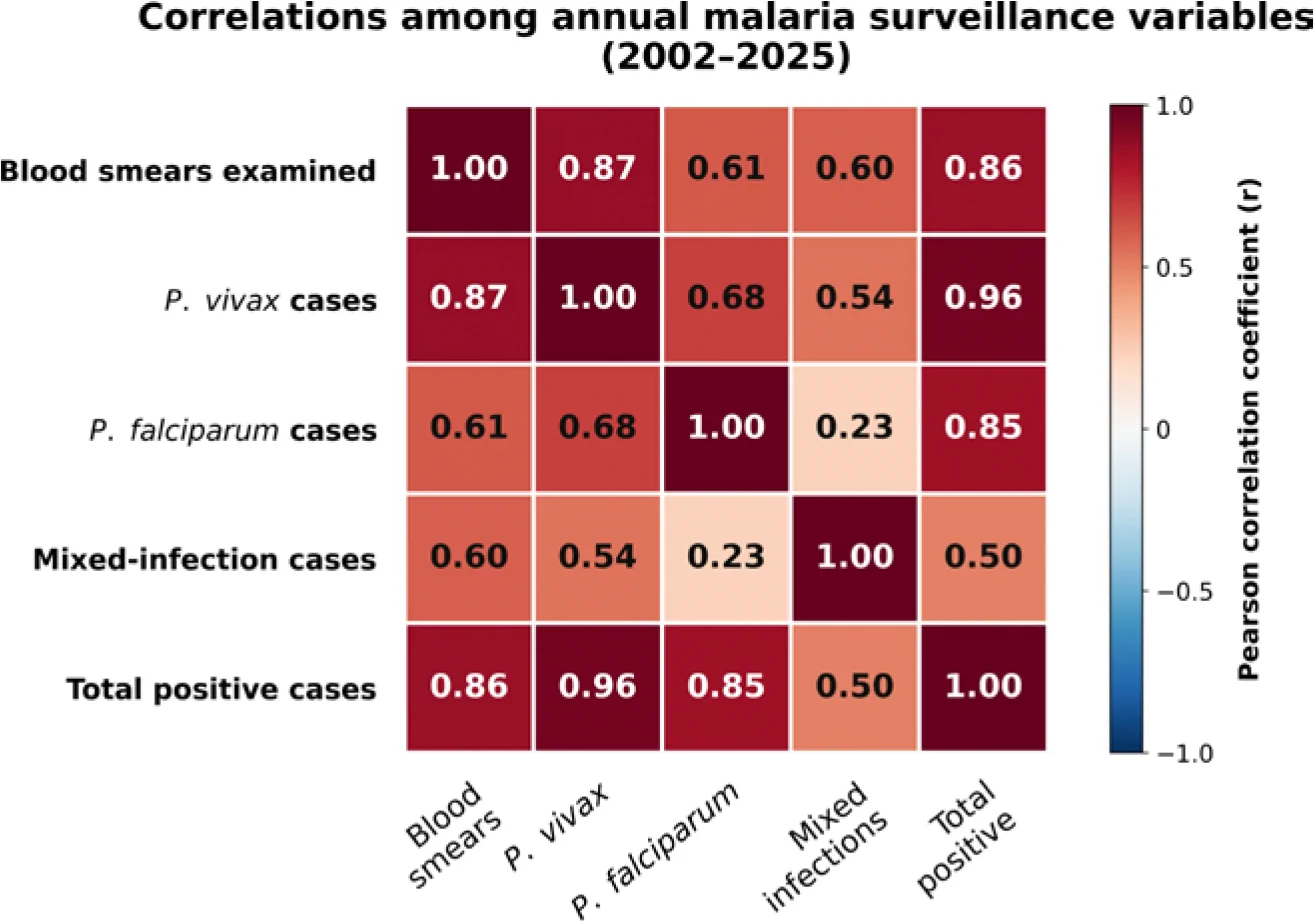
Correlations Among Annual Malaria Surveillance Variables, 2002–2025. The heatmap presents Pearson correlation coefficients (*r*) among the annual numbers of blood smears examined, *Plasmodium vivax* cases, *P. falciparum* cases, mixed infections, and total malaria-positive cases recorded at Wenlock District Government Hospital, Mangaluru, India. Values within the cells represent correlation coefficients, with values approaching +1 indicating stronger positive correlations, values approaching −1 indicating stronger negative correlations, and values near zero indicating weak linear relationships. Red represents positive correlations, blue represents negative correlations, and lighter colours represent coefficients closer to zero. The strongest observed correlation was between *P. vivax* cases and total malaria-positive cases (*r* = 0.96), whereas the weakest was between *P. falciparum* cases and mixed infections (*r* = 0.23). Diagonal values of 1.00 represent the correlation of each variable with itself.

**Table 1:** Annual Distribution of Confirmed Malaria Cases at Wenlock Hospital, Mangaluru, 2002–2025

Year	Blood smears examined, n	P. vivax, n (%)	P. falciparum, n (%)	Mixed infection, n (%)	Total positive, n (%)
**2002**	10,417	1,994 (77.07)	587 (22.69)	6 [0.23]	2,587 (24.83)
**2003**	13,225	2,694 (76.59)	810 (23.03)	13 (0.36)	3,517 (26.59)
**2004**	18,872	4,773 (64.12)	2,645 (35.53)	25 (0.33)	7,443 (39.43)
**2005**	22,219	4,346 (53.39)	3,675 (45.15)	118 (1.44)	8,139 (36.63)
**2006**	20,481	3,895 (56.28)	2,832 (40.92)	193 (2.78)	6,920 (33.78)
**2007**	15,273	2,640 (63.40)	1,443 (34.65)	81 (1.94)	4,164 (27.26)
**2008**	16,266	2,810 (77.62)	717 (19.80)	93 (2.53)	3,620 (22.25)
**2008**	17,659	3,259 (71.84)	1,180 (26.01)	97 (2.14)	4,536 (25.68)
**2010**	15,445	3,175 (77.49)	853 (20.82)	69 (1.68)	4,097 (26.52)
**2011**	17,542	3,855 (84.43)	658 (14.41)	53 [1.16]	4,566 (26.02)
**2012**	18,441	3,911 (86.14)	564 (12.42)	65 (1.43)	4,540 (24.61)
**2013**	19,490	3,994 (86.82)	512 (11.13)	94 (2.04)	4,600 (23.60)
**2014**	23,123	5,422 (80.12)	995 (14.70)	350 (5.17)	6,767 (29.26)
**2015**	20,097	3,305 (74.69)	609 (13.76)	511 (11.55)	4,425 (22.01)
**2016**	17.296	2,613 (83.11)	340 (10.81)	191 (6.07)	3,144 (18.17)
**2017**	14,286	1,676 (80.49)	346 (16.62)	60 (2.88)	2,082 (14.57)
**2018**	11.767	1,159 (79.27)	273 (18.67)	30 (2.05)	1,462 (12.42)
**2018**	13,651	657 (75.60)	186 (21.40)	16 [1.84]	859 (6.29)
**2020**	2,425	184 (81.05)	38 (16.74)	5 (2.20)	227 (9.36)
**2021**	3,423	166 (68.03)	63 (25.81)	15 (6.14)	244 (7.12)
**2022**	5,812	40 (86.95)	6 (13.04)	0 [0.00]	46 (0.79)
**2023**	12,320	8 (61.53)	5 (38.46)	0 [0.00]	13 (0.10)
**2024**	12,612	37 (86.04)	5 (11.62)	1 (2.32)	43 (0.34)
**2025**	3,402	8 (66.66)	4 (33.33)	0 [0.00]	12 (0.35)
**Overall**	345,544	56,621 (72.54)	19,346 (24.78)	2,086 (2.67)	78,053 (22.58)

**Note**: Values are n (%) unless otherwise indicated. Percentages for P. vivax, P. falciparum, and mixed infections were calculated among malaria-positive cases; total-positive percentages were calculated among blood smears examined.

**Table 2:** Associations Between Malaria Infection Group and Clinical Characteristics

Clinical characteristic	Pearson χ^2^	df	P value	Cramer’s V	Effect size
**Fever**	3.540	2	0.170317	0.0067	Negligible
**Headache**	14.020	2	0.000903	0.0134	Negligible
**Chills**	1,697.146	2	<0.000001	0.1475	Weak
**Shivering**	14,262.081	2	<0.000001	0.4275	Strong
**Sweating**	173.333	2	2.30×10^−38^	0.0471	Negligible
**Cough**	7,729.552	2	<0.000001	0.3147	Moderate
**Runny nose**	3,065.854	2	<0.000001	0.1982	Weak
**Nausea**	8,215.259	2	<0.000001	0.3244	Moderate
**Vomiting**	6,388.693	2	<0.000001	0.2861	Moderate
**Sore throat**	118.264	2	2.09×10^−26^	0.0389	Negligible
**Bitter mouth**	195.319	2	3.86×10^−43^	0.0500	Negligible
**Fatigue**	1,875.525	2	<0.000001	0.1550	Weak
**Dizziness**	116.385	2	5.34×10^−26^	0.0386	Negligible
**Abdominal pain**	750.857	2	8.98×10^−164^	0.0981	Negligible
**Diarrhoea**	408.139	2	2.36×10^−89^	0.0723	Negligible
**Loss of appetite/anorexia**	8,708.541	2	<0.000001	0.3340	Moderate
**Myalgia (back and limbs)**	7,993.436	2	<0.000001	0.3200	Moderate
**Breathing difficulty**	8,433.084	2	<0.000001	0.3287	Moderate
**Burning sensation in the eyes**	1,906.381	2	<0.000001	0.1563	Weak
**Burning sensation in the chest**	5,355.252	2	<0.000001	0.2619	Moderate
**Hypertension**	18,376.143	2	<0.000001	0.4852	Strong

**Note:** All Pearson chi-square tests had 2 degrees of freedom. Cramer’s V was used to classify the strength of association. P < 0.05 was considered statistically significant. Strong associations with infection group were observed for shivering and hypertension. Moderate associations were observed for cough, nausea, vomiting, loss of appetite/anorexia, myalgia, breathing difficulty, and burning sensation in the chest; weak associations were observed for chills, runny nose, fatigue, and burning sensation in the eyes. The remaining characteristics showed negligible associations. Twenty-one binary clinical characteristics were compared across three malaria infection groups: P. vivax (n = 56,621), P. falciparum (n = 19,346), and mixed infection (n = 2,086), for a combined total of 78,053 cases. Associations were evaluated using 3 x 2 Pearson chi-square tests (symptom present/absent x infection group), and Cramer’s V quantified effect size. Tests were two-sided, with P < 0.05 considered statistically significant.

**Table 3:** Bonferroni-adjusted Pairwise Comparisons Among Malaria Infection Groups

Clinical characteristic	P. vivax vs P. falciparum, adjusted P	P. vivax vs mixed infection, adjusted P	P. falciparum vs mixed infection, adjusted P
**Fever**	1.0000	0.3590	0.2347
**Headache**	<0.001	1.0000	0.0884
**Chills**	<0.001	<0.001	<0.001
**Shivering**	<0.001	<0.001	<0.001
**Sweating**	<0.001	<0.001	0.0029
**Cough**	<0.001	<0.001	<0.001
**Runny nose**	<0.001	<0.001	0.0065
**Nausea**	<0.001	<0.001	0.0116
**Vomiting**	<0.001	<0.001	<0.001
**Sore throat**	<0.001	<0.001	<0.001
**Bitter mouth**	<0.001	0.0327	<0.001
**Fatiguo**	<0.001	<0.001	1.0000
**Dizziness**	<0.001	0.3960	0.0459
**Abdominal pain**	<0.001	<0.001	0.0050
**Diarrhoea**	<0.001	<0.001	1.0000
**Loss of appetite/ anorexia**	<0.001	<0.001	<0.001
**Myalgia**	<0.001	<0.001	0.0014
**Breathing difficulty**	<0.001	<0.001	<0.001
**Burning sensation in eyes**	<0.001	<0.001	<0.001
**Burning sensation in chest**	<0.001	<0.001	<0.001
**Hypertension**	<0.001	<0.001	<0.001

**Note:** Values are Bonferroni-adjusted P values from pairwise Pearson chi-square tests. The correction accounts for three comparisons within each clinical characteristic; adjusted P < 0.05 was considered statistically significant. After Bonferroni correction, 55 of 63 pairwise comparisons remained statistically significant (adjusted P < 0.05).

## Data Availability

The datasets generated and/or analyzed during the current study are archived. Source data used for comparative or illustrative purposes are uniquely identified and available from the corresponding author upon reasonable request.

## Abbreviations

MMC: monthly malaria cases
ETF: early treatment failure
LTF: late treatment failure
MDA: mass drug administration
ACT: artesunate + sulfadoxine + pyrimethamine
Pfcrt: Plasmodium falciparum chloroquine resistance transporter
Pfmdr1: Plasmodium falciparum multidrug resistance protein 1
PfK13: Plasmodium falciparum kelch 13 gene
nested PCR: nested polymerase chain reaction
qPCR: quantitative polymerase chain reaction
LLINs: long-lasting insecticidal nets
IRS: indoor residual spraying
ITN: insecticide-treated net
AI: Artificial intelligence
HIV: human immunodeficiency virus
AIDS: acquired immune deficiency syndrome
WHO-SEARO: World Health Organization-South-East Asia Region
